# Effect Of Transtibial Prosthesis Mass On Gait Asymmetries

**DOI:** 10.33137/cpoj.v3i2.34609

**Published:** 2020-10-19

**Authors:** M. Seth, W. Hou, L.R. Goyarts, J.P. Galassi, E.M. Lamberg

**Affiliations:** 1 School of Health Technology and Management, Stony Brook University, Stony Brook, NY, USA.; 2 School of Medicine, Stony Brook University, Stony Brook, NY, USA.

**Keywords:** Prosthesis, Mass, Gait, Lower limb amputation, Kinematics, Kinetics, Ground reaction forces, Transtibial amputation, Gait asymmetry

## Abstract

**BACKGROUND:**

Individuals with transtibial amputation (TTA) typically walk with an asymmetrical gait pattern, which may predispose them to secondary complications and increase risk of fall. Gait asymmetry may be influenced by prosthesis mass.

**OBJECTIVES:**

To explore the effects of prosthesis mass on temporal and limb loading asymmetry in people with TTA following seven days of acclimation and community use.

**METHODOLOGY:**

Eight individuals with transtibial amputation participated. A counterbalanced repeated measures study, involving three sessions (each one week apart) was conducted, during which three load conditions were examined: no load, light load and heavy load. The light load and heavy load conditions were achieved by adding 30% and 50% of the mass difference between legs, at a proximal location on the prosthesis. Kinematic and ground reaction force data was captured while walking one week after the added mass. Symmetry indices between the prosthetic and intact side were computed for temporal (Stance and Swing time) and limb loading measures (vertical ground reaction force Peak and Impulse).

**FINDINGS:**

Following seven days of acclimation, no significant differences were observed between the three mass conditions (no load, light load and heavy load) for temporal (Stance time: p=0.61; Swing time: p=0.13) and limb loading asymmetry (vertical ground reaction force Peak: p=0.95; vertical ground reaction force Impulse: p=0.55).

**CONCLUSIONS:**

Prosthesis mass increase at a proximal location did not increase temporal and limb loading asymmetry during walking in individuals with TTA. Hence, mass increase subsequent to replacing proximally located prosthesis components may not increase gait asymmetry, thereby allowing more flexibility to the clinician for component selection.

## INTRODUCTION

Individuals living with a transtibial amputation (TTA) typically present with an asymmetrical gait pattern characterized by a prolonged stance phase (temporal asymmetry)^1-5^ and greater loading on the intact side as compared to the prosthetic side (limb loading asymmetry).^5-8^ A prolonged asymmetrical gait may predispose the individual to poor health outcomes, such as, knee or hip osteoarthritis of the intact side,^8,9^ back pain,^10^ and an increased risk of falling.^11^ Further, walking with an asymmetrical gait pattern may attract unwanted attention towards the individual.^12^ While, inherent factors associated with a TTA, such as weak prosthetic side push-off force ^13^ and issues pertaining to load bearing of the residual limb,^14^ may contribute to gait asymmetry, certain prosthetic factors, such as mass, may also contribute to gait asymmetry. Currently, the impact of prosthesis mass on gait asymmetry is not well defined. Further exploration may be vital to identifying any potential negative consequences that prosthesis mass may have on gait asymmetry and by extension health outcomes, of adults with TTA.

Previous efforts to determine the relationship between prosthesis mass and gait asymmetry have been completed, but only with limited application. In one mathematical study, it was theorized that temporal asymmetry seen in the gait of adults with lower limb amputation may be minimized by the achievement of inertial symmetry between the intact and prosthetic side.^15^ Other experimental and mathematical studies, however, found that matching prosthetic side mass completely (100%) with the intact side, through the addition of a distal load, produced an increase in temporal asymmetry.^16-19^ Thus, suggesting that a prosthesis should not be as heavy as the segment it replaces, particularly if mass is distributed more distally. However, the experimental evidence is limited regarding smaller prosthesis mass increments (<100%) and the application of mass at proximal locations. This would result in a prosthesis heavier than its original weight, but lighter in comparison to the intact side. Mass alterations at proximal locations are common clinically, subsequent to changing prosthesis components, i.e., socket, liner or socket/pylon interface adapters. Hence, it is important to understand if these types of common increases in proximal prosthetic mass effect temporal asymmetry during walking.

Further, the relationship between prosthesis mass and limb loading in people with TTA during walking has undergone limited exploration. Using a single subject design Hillary et al. examined the effects of increasing prosthesis mass by 0.53kg and 1.46kg on limb loading in an adult with TTA.^20^ The increase in prosthesis mass resulted in higher limb loading forces (1st vertical ground reaction force peak) on both the prosthetic and intact side.^20^ These increased impact forces are concerning as they may have consequences for bone and joint health.^8,9^

Increasing prosthesis mass, through proximal load application, will make a prosthesis heavier, but may not always make the prosthetic side as heavy as the intact side (or 100% matching). Currently, it is unclear whether such mass increments to a prosthesis, i.e., less than 100% matching, will influence gait temporal and loading asymmetry of individuals with TTA. Further, the majority of current evidence on prosthesis mass is based on short acclimation periods prior to experimental data collection, which may yield differing results. The purpose of this study is to explore the effects of increasing prosthesis mass on temporal and limb loading asymmetry in people with TTA following seven days of acclimation and community use.

## METHODOLOGY

A counterbalanced repeated measures study was conducted to examine the temporal and limb loading asymmetry in adults with TTA under three load conditions: original prosthesis mass with no load added (No load; NL), original prosthesis mass with 30% of the mass difference between prosthetic and intact side added (Light load; LL), and original prosthesis mass with 50% of the mass difference between prosthetic and intact side added (Heavy load; HL). The choice of the mass conditions was based on a pilot unpublished retrospective analysis of 12 medical charts of individuals with TTA. On an average, 30% (450g) and 50% (750g) of the mass difference between the prosthetic and intact side may represent the approximate mass change that may happen as a result of changing common prosthesis components.

### Participants

Participants were included if they were male or female, between the ages of 18 to 70 years, had a unilateral amputation ≥ one year prior to participation and were community ambulators. Further, to minimize potential effects due to recent changes in prosthesis or any components, only individuals using their current prosthesis for at least three months were included. Potential participants were excluded if they had a health condition (cardiac, pulmonary, or musculoskeletal) limiting their ability to walk or if their prosthetic side was heavier than their intact side. Based on previous literature on this topic,^16,17,19^ the study was planned to enroll a minimum of 6 individuals with TTA. Informed written consent was obtained before participation. The study was approved by Stony Brook University’s Institutional Review Board.

### Limb mass properties

Body mass (kg), height (m), residual limb and prosthesis measurements were ascertained. Prosthetic side mass (PSM) was calculated as the sum of prosthesis mass (with liner and shoe on a scale) and residual limb mass. To calculate residual limb mass, the residual limb was mathematically modeled as the frustum of a right circular cone using circumferential and length measurements as inputs and assuming a uniform tissue density of 1.1gm/cm3.^21^ In order to calculate intact side mass, body mass (BM) needed to be adjusted to account for the amputated limb segment^17^:


Equation 1: $$ABM=\frac{BM-PSM}{\left(1-c\right)}$$

Where, BM is measured body mass while wearing the prosthesis, PSM is prosthetic side mass, and c is the amount of mass accounted by an intact shank and foot (0.057 for men and 0.061 for women).^17^ Then, using ABM the intact side shank and foot mass was calculated from standard limb segment mass estimates (5.7% for men and 6.1% for women).^22^ In order to determine the load amount for each participant, the difference between prosthetic side and intact side mass was calculated and 30% (LL condition) and 50% (HL condition) of that difference was identified.

### Research protocol

**Figure 1:**
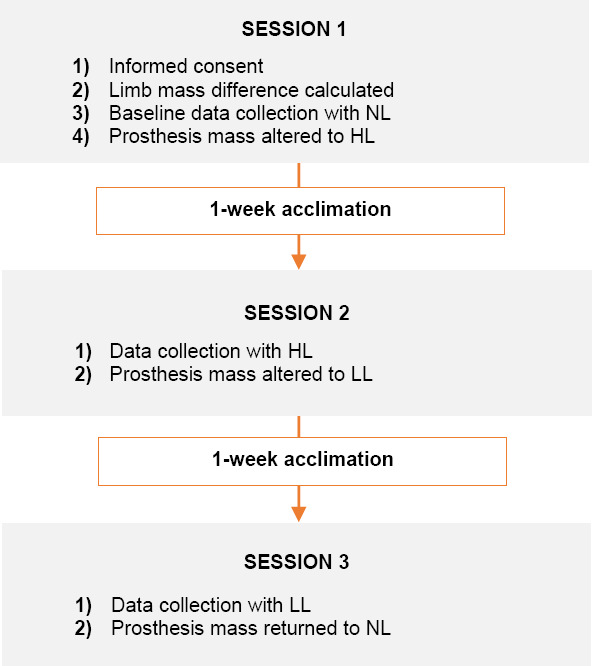
Timeline of research sessions for one participant. The order of HL or LL was counterbalanced so that half received HL at session 1 then LL at session 2 and half received LL at session 1 then HL at session 2. NL= No load, LL= Light load, HL= Heavy load.

Participants attended three testing sessions scheduled one week apart (**Figure 1**), during which the load conditions (NL, LL and HL) were examined. During session one, study purpose and procedures were explained and the mass difference between limbs was calculated. Participants were then prepared for baseline data collection with their original prosthesis mass (NL). Following NL data collection, prosthesis mass was altered by adding either 30% (LL) or 50% (HL) of the mass difference between limbs. Mass was added circumferentially to the prosthesis socket at a proximal location that was 20 to 30% of the non-prosthetic side length. Flexible car wheel balancing weights (BADA steel tape-a-weight, Hennessey Industries LLC, LaVergne, TN, USA) were affixed at the desired location with one sided tape that was further held in place by wrapping Coban (3M™, Saint Paul, MN, USA) to prevent any loosening or slippage. The load sequence was altered between participants such that half received HL then LL and half received LL then HL. Once the mass was altered participants went home to use their prosthesis for seven days during their daily activities.

### Data collection

For all sessions, participants were prepared with the application of passive reflective markers over standardizedanatomical landmarks on their pelvis and lower limbs using the modified Hellen Hayes marker set-up.^23^ On the prosthesis, markers were placed at locations corresponding to the intact side. Participants walked at their comfortable walking speed over a 10m walkway, while kinematic data was captured using a ten camera Vicon Motion System^®^ (Oxford Metrics, Oxford, UK), recording at 100 Hz. In addition, ground reaction force (GRF) was captured through AMTI^®^ force-plates (Watertown, USA) recording at 1000Hz.

### Data analysis

Customized scripts developed in Matlab^®^ (Mathworks, Inc.), were used to analyze and process the temporal and GRF data. Gait events (heel strike and toe off) were identified from the raw marker data extracted from Nexus VICON using the methodology proposed by Zeni et al.^24^ Temporal measures of stance time (heel strike to toe off) and swing time (toe off to heel strike) were determined for each leg. GRF data was processed using a zero-lag low-pass 4th order Butterworth filter, with a cutoff frequency of 20Hz.

The beginning and end of stance phase for the vertical component of GRF (vGRF) was established using a vGRF threshold of 20N.^25^ The vGRF was normalized to each participant’s body mass (with prosthesis) and subsequently the maximum value during the 1st half of the stance phase was identified (vGRF Peak) and the area under the curve throughout the entire stance phase was calculated (vGRF Impulse).

Symmetry indices (SI) for temporal and loading measures were calculated for each load condition^26^ where, I and P refer to intact and prosthetic side values. The SI ranges between 0 and 1, where 0 represents perfect symmetry. A positive SI indicates a higher value for the intact side:


Equation 2: $$SI=\frac{\left(I-P\right)}{(I+P)*0.5}$$

### Statistics

A single-factor repeated measures analysis of variance (ANOVA) was used to test for differences in SI for stance time, swing time, vGRF Peak and vGRF Impulse among the NL, LL, and HL conditions. An alpha level of 0.05 was used to evaluate significance.

## RESULTS

### Study Sample

Eight male individuals with unilateral TTA who ambulate without assistive devices participated in this study (**Table 1**). Overall, 19 inquiries were received, and 14 individuals were screened over the phone. Of these 14, nine consented while five did not meet inclusion criteria or stated they did not want to add load to their device. Of the nine consented, one was subsequently excluded because the prosthetic side mass was higher than the intact side mass. **Table 2** presents the mean of the calculated mass values.

**Table 1:** Participant demographics and clinical characteristics.

Participant	Age (yrs)	Body mass (kg)^*^	Height (m)	Time since amputation (yrs)	Amputation etiology	Side
01	48	85.0	1.88	6	Infection	Left
02	29	101.0	1.72	4	Neurofibromatosis	Left
03	50	113.1	1.86	1	Trauma	Left
04	62	117.5	1.83	2	Vascular	Right
05	26	95.0	1.84	14	Cancer	Left
06	57	91.6	1.81	7	Trauma	Left
07	30	75.0	1.79	30	Congenital	Right
08	59	88.5	1.76	59	Congenital	Right
**Mean**	**45.1**	**95.8**	**1.81**	**15.4**	**-**	**-**
**(SD)**	**(14.7)**	**(14.2)**	**(0.05)**	**(20)**		

* *Body mass was measured with the prosthesis and shoe on the participant.*

**Table 2:** Estimated mass properties of the prosthetic and intact side.

Measure		Mean (SD)	Range
Adjusted body mass		96.9 (14.8)	75.4 – 119.5
Non-prosthetic side mass		5.5 (0.8)	5.3 – 6.8
Prosthetic side mass		4.5 (0.4)	3.9 – 5.2
Mass difference between sides		1.0 (0.7)	0.4 – 2.3
**Load conditions**	Light Load (30% of difference)	0.3 (0.2)	0.1 – 0.7
	Heavy Load (50% of difference)	0.5 (0.3)	0.2 – 1.1

*All values in kg. Non-prosthetic side mass refers to estimated shank and foot mass of non-prosthetic side. Prosthetic side mass refers to the sum of estimated residual limb mass and prosthesis mass (with shoe, liner and suspension system).*

### Main results

**Figure 2:**
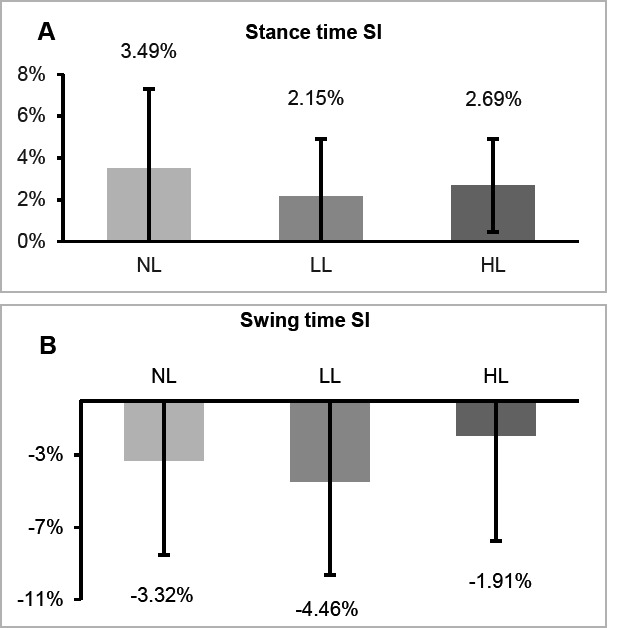
Mean stance time SI (A) and swing time SI (B) across the three load conditions. Error bars represent ±1 SD. Negative SI values indicate that intact side values were higher. NL= No load, LL= Light load, HL= Heavy load.

**Figure 3:**
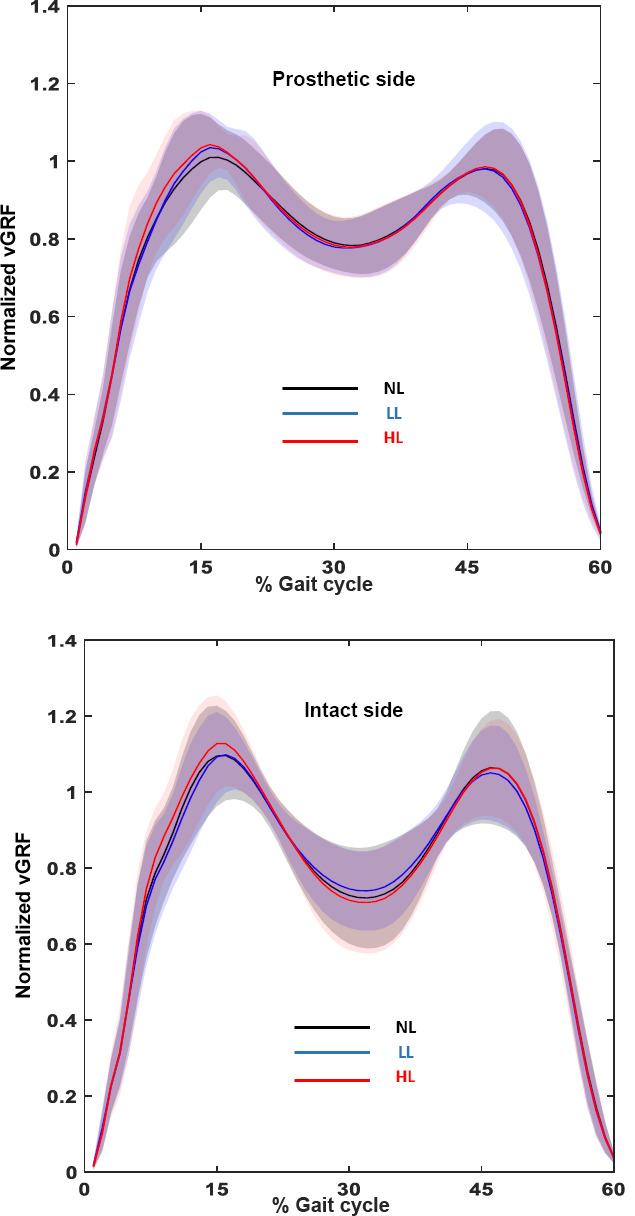
Mean normalized vGRF activity of the prosthetic (A) and intact side (B) across the three load conditions (NL, LL and HL). The shaded region represents 1±SD of the group mean^27^ for that load condition. NL= No load, LL= Light load, HL= Heavy load.

The mean (SD) for stance time, swing time, vGRF Peak and vGRF Impulse on the prosthetic and intact side with the three load conditions (NL, LL and HL) and the SI are presented in **Table 3**. The mean (SD) stance time SI and swing time SI for the three load conditions is also presented in **Figure 2**. The mean vGRF profile of the participants for the prosthetic and intact side with the three load conditions (NL, LL and HL) is presented in **Figure 3**.

**Table 3:** Mean (SD) temporal and GRF measures for the three load conditions. NL= No load, LL= Light load, HL= Heavy load.

	NL			LL			HL			Sig.(*p-value*)
	P	NP	SI%	P	NP	SI%	P	NP	SI%	
**Stance time (secs)**	0.77	0.80	3.49%	0.79	0.80	2.15%	0.76	0.78	2.69%	0.61
	(0.04)	(0.05)	(3.79)	(0.04)	(0.05)	(2.77)	(0.05)	(0.05)	(2.23)	
**Swing time (secs)**	0.40	0.39	-3.32%	0.41	0.39	-4.46%	0.40	0.39	-1.91%	0.13
	(0.03)	(0.02)	(5.23)	(0.01)	(0.02)	(5.16)	(0.02)	(0.02)	(5.86)	
**vGRF Peak (BM)**	1.03	1.14	9.70%	1.05	1.15	9.06%	1.06	1.16	9.51%	0.95
	(0.07)	(0.12)	(10.70)	(0.07)	(0.07)	(6.20)	(0.08)	(0.09)	(9.35)	
**vGRF Impulse (BM)**	0.56	0.60	7.99%	0.57	0.61	6.89%	0.56	0.60	6.73%	0.55
	(0.04)	(0.04)	(7.11)	(0.03)	(0.04)	(6.32)	(0.04)	(0.03)	(7.53)	

*P: mean (SD) raw score for the prosthetic side; NP: mean (SD) raw score for the non-prosthetic side; SI%: symmetry index % between the prosthetic and non-prosthetic side; BM: normalized to body mass. The p-values between the three load conditions are for the SI%*

Following seven days of acclimation, increasing the mass of the prosthesis by 30% and 50% did not significantly affect the SI values between the prosthetic and intact side for temporal (Stance time: p=0.61; swing time: p=0.13) and loading measures (vGRF Peak: p=0.95; vGRF Impulse: p=0.55).

## DISCUSSION

This study explored the impact of increasing prosthesis mass on temporal and limb loading asymmetry in people with TTA following seven days of acclimation and community use. According to the findings, increasing prosthesis mass by either 30% or 50% of the mass difference between legs did not significantly alter temporal and limb loading asymmetry. These findings imply that a range of prosthesis mass may exist for this population. This ultimately may allow clinicians flexibility when choosing prosthesis components knowing that increasing mass within this range may not negatively impact gait asymmetries.

### Temporal asymmetry

Literature on transtibial prosthesis mass has largely focused on examining temporal asymmetry during walking.^15-19^ Perhaps the proposed association between limb inertia and swing time or the secondary complications as a result of an asymmetrical gait, necessitate the examination of temporal measures following mass alterations. In this study, stance and swing time asymmetries were not significantly affected as a result of the mass alteration. Previous studies examining stance and swing time asymmetries have found that matching prosthetic side mass to the intact side (100% load condition) increases asymmetry.^16-19^ The same however, is not true for partial matching (50% load condition). Similar to the findings of the current study, Smith and Martin report no effect of the 50% load condition on stance and swing time asymmetry.^17^ However, Mattes et al. observed a significant increase in temporal asymmetry with the 50% load condition.^16^ Hence, Mattes et al., observed a linear response to the mass increase; as prosthesis mass increased, stance time on the intact side and swing time on the prosthetic side increased, thereby increasing limb temporal asymmetry.^16^ Interestingly, Mattes et al., used a similar approach as the current study to estimate mass difference between limbs. However, they achieved the 50% condition by adding an average load of 0.85kgs.^16^ while this study used an average of 0.54kgs, which may partially explain the divergence in findings. Perhaps the key difference between the studies is the length of acclimation period used, ten minutes by Mattes et al.,^16^ versus one week in this study. The longer acclimation time provided in this study may have allowed participants to stabilize their gait, leading to the lack of statistically significant findings. Although, in previous research it has been observed that stance and swing time asymmetries (as a result of the 100% load condition) persisted even after eight days of acclimation^19^; this finding, doesn’t necessarily rule out the importance of acclimation. It is more likely that individuals with TTA are not well suited to carry a prosthesis that weighs as much as the amputated segment.

### Limb loading asymmetry

Limb loading asymmetry (measured using vGRF Peak and vGRF Impulse SI), was not affected as a result of the mass increase, i.e., no differences were observed between the three load conditions (NL, LL and HL). The positive SI values (**Table 3**) indicate that participants in this study loaded their intact side more than the prosthetic side, consistent with previous findings in the literature.^5-8^ The addition of load, however, did not significantly change the proportion of loading experienced by the intact side.

In the single subject case study by Hillery et al., the authors observed an increase in loading (vGRF Peak) on the prosthetic and the intact side with an increase in prosthesis mass.^20^ Based on the SI equation,^26^ it appears that the study participant’s loading asymmetry increased from 5.4% at baseline to 12% with the intermediate load (0.5kgs) and 21% with the heaviest load (1.5kgs).^20^ In contrast, vGRF Peak SI in the current study changed from an average of 9.70% at baseline to 9.1% with LL (0.3kgs) and 9.5% with the heaviest load condition, HL (0.5kg). This difference in findings may be due to the heavier loads used by Hillery et al., or perhaps the longer acclimation period given in the current study allowed users to stabilize their gait before data was collected. However, given the single-subject study design by Hillery et al., their findings may not be generalized to a larger group.^20^

In a more recent study of a group of active adults with TTA (n=10), Alcantara et al. examined the impact of increasing the mass of a running-specific prosthesis and biological foot on participants’ limb loading asymmetry during running.^28^ Prosthesis and biological foot mass were increased by adding 100g or 300g at the toe region. The mass increase, however, did not result in any significant change to limb loading asymmetry (vGRF peak SI or average-stance vGRF SI) as compared to baseline, regardless of added load (100g or 300g) or whether mass was added to the prosthesis or both limbs.^28^ Obvious differences exist between Alcantara et al., and the current study, such as the location of mass addition or their use of a running task. However, collectively, these findings suggest that small increases in prosthesis mass may not contribute to the limb loading asymmetry and by extension lower health outcomes generally associated with asymmetrical limb loading, such as knee or hip osteoarthritis of the intact side.

The need to change a prosthesis component or replace a prosthesis completely may arise due to various personal and/or clinical reasons. Such changes may increase or decrease the overall prosthesis mass. For example, the replacement of prefabricated transtibial gel liners, which range from 202g to 722g, may alter prosthesis mass.^29^ Findings from this study suggest that the mass increments similar to those observed after replacing proximally located prosthesis components (such as, the socket, liners, or socket/pylon interface adapters), may not increase gait asymmetry. These findings are clinically relevant as they may serve as a guide for clinicians when routinely adjusting prosthesis mass. However, to further identify the impact of prosthesis mass on gait asymmetry, it is vital that future large-scale studies explore the impact of various commercially available prosthesis components and related functional and clinical outcomes. Moreover, future studies may consider controlling other factors that may influence gait characteristics of an individual, for example, residual limb length, quality and type of suspension between the prosthesis and residual limb, or exertion of the individual during a walking task.

### Study limitations

The current study was limited both by a small sample size (n=8), and a male-only participant group. It is possible that the small sample size may not have had sufficient power to detect a significant change. Moreover, in order to make stronger conclusions on prosthesis mass, it remains necessary to evaluate both men and women with TTA. As part of the inclusion criteria, only individuals that were capable of independent ambulation were included within the study. Therefore, the current findings may not extend to individuals with TTA who exhibit lower levels of activity. Further, this study chose to limit mass increase to 50% of the mass difference between both limbs. To establish the upper limit of a TTP mass it may be necessary to evaluate heavier loads. Lastly, certain covariates, such as, residual limb length, quality and type of suspension between the prosthesis and residual limb or prosthesis componentry may have impacted both gait asymmetry and prosthesis mass of the study participants but were not controlled in this study.

## CONCLUSION

Temporal and loading asymmetries during walking in people with TTA may lead to secondary complications of the intact side and increase risk of falls. Currently, it is unclear if prosthesis mass alterations that may occur during routine clinical visits will impact these gait asymmetries. This exploratory study observed that following seven days of use increases in proximally distributed prosthesis mass (up to 50% of the mass difference between legs), did not further increase temporal and limb loading asymmetry of individuals with TTA, thereby allowing more flexibility to the clinician for component selection. Confirmation of findings with large-scale studies may help identify the impact of prosthesis mass on gait asymmetry.

## DECLARATION OF CONFLICTING INTERESTS

The authors have no conflicts of interests to declare.

## AUTHOR CONTRIBUTION

**Mayank Seth:** Study conceptualization, analysis, data collection, data interpretation and writing - original draft

**Wei Hou:** Assisted in study design, led statistical analysis, writing - review & editing

**Laura R Goyarts:** Assisted in data collection, writing - review & editing

**James P Galassi:** Assisted in data collection, writing - review & editing

**Eric M Lamberg:** Study conceptualization, project administration, supervision, data interpretation, writing – review & editing

## SOURCES OF SUPPORT

This research did not receive any specific grant from funding agencies in the public, commercial or not-for-profit sectors.

## ETHICAL APPROVAL

The study was approved by Stony Brook University’s Institutional Review Board.
